# Development and validation of a sequential two-step algorithm for the screening of individuals with potential polycythaemia vera

**DOI:** 10.1038/s41598-020-80459-y

**Published:** 2021-01-08

**Authors:** Miguel Piris-Villaespesa, Alberto Álvarez-Larrán, Adolfo Saez-Marín, Claudia Nuñez-Torrón, Gloria Muñoz-Martin, Ricardo Sánchez, Francisco J. del Castillo, Jesús Villarrubia, Javier Lopez-Jimenez, Joaquin Martinez-Lopez, Valentin Garcia-Gutierrez

**Affiliations:** 1 Haematology Department, Hospital Universitario Ramón Y Cajal, Madrid, Spain; 2 Instituto Ramón Y Cajal de Investigación Sanitaria (IRYCIS), Madrid, Spain; 3 Haematology Department, Hospital Clínic, IDIBAPS, Barcelona, Spain; 4 Translational Genomics Unit, Hospital Universitario Ramón Y Cajal (IRYCIS), Madrid, Spain; 5 Haematology Department, Hospital Universitario 12 de Octubre, Madrid, Spain; 6 Genetics Department, Hospital Universitario Ramón Y Cajal, Madrid, Spain; 7 Centro de Investigación Biomédica en Red de Enfermedades Raras (CIBERER), Madrid, Spain; 8 Complutense University of Madrid, Madrid, Spain; 9 Centro Nacional de Investigaciones Oncológicas, Madrid, Spain

**Keywords:** Molecular medicine, Oncology, Cancer, Cancer genetics, Cancer screening, Haematological cancer, Biomarkers, Diagnostic markers, Diagnostic markers, Medical research, Translational research

## Abstract

In 2016, the WHO included haemoglobin values within normal ranges as a diagnostic criterion for Polycythaemia Vera (PV). Since then, concerns have arisen that a large number of patients are undergoing unnecessary screening for PV. To address this issue, we estimated the prevalence of *JAK2* p.V617F in individuals with elevated haemoglobin or haematocrit and developed and validated a screening algorithm for PV. A total of 15,366 blood counts performed in seven non-consecutive days were reviewed, of which 1001 were selected for subsequent *JAK2* p.V617F mutation screening. Eight (0.8%) new *JAK2* p.V617F-mutated cases were detected. From ROC curves, a two-step algorithm was developed based on the optimal cut-off for the detection of the *JAK2* p.V617F mutation. The algorithm was prospectively validated in an independent cohort of 15,298 blood counts. A total of 1595 (10.4%) cases met the criterion for haemoglobin or haematocrit, of whom 581 passed to step 2 (3.8% of the total). The *JAK2* p.V617F mutation was detected in 7 of the 501 patients tested, which accounts for 0.04% of the total cohort and 0.4% of patients with erythrocytosis. In conclusion, this data show that our two-step algorithm improves the selection of candidates for *JAK2* p.V617F testing.

## Introduction

The presence of the *JAK2* p.V617F mutation one of the major criterion for the diagnosis of Polycythaemia Vera (PV), although it is found in other myeloproliferative neoplasms^1^. Another major diagnostic criterion is elevated haemoglobin (Hb) concentration (> 16.5 g/dL in men; > 16 g/dL in women) or elevated haematocrit (Htc) (> 49% in men; > 48% in women) or increased red blood cell mass^1^. The thresholds for haemoglobin and haematocrit were modified in the 2016 World Health Organization (WHO) update. These thresholds were modified after it was observed that 40% of cases of PV were misdiagnosed when the 2008 WHO Hb thresholds were used and red cell mass or bone marrow biopsy was not performed^2^. However, some studies suggest that the 2016 WHO haemoglobin thresholds could lead to unnecessary testing. Thus, with these thresholds, the number of men and women tested has been calculated to have increased 12-fold and threefold, respectively, which also has a high psychosocial burden^3,4^. In order to provide a solution, some authors recommend that higher haemoglobin cut-off values are used, or that the diagnostic algorithm is based on the combined presence of thrombocytosis, neutrophilia or previous thrombosis^3,5^. Nonetheless, even when these diagnostic approaches are applied, a high proportion of cases will be still missed, with a high rate of false negatives (14–64%)^6^.

Several recent reports highlight the relevance of detecting *JAK2* p.V617F clonal haematopoiesis, since it is strongly associated with an increased cardiovascular risk^7,8^. In large cohort studies, the prevalence of *JAK2* p.V617F in the general population has been estimated to range between 0.1 and 3.1%. This variability can be explained by differences in the sensitivity of the tests used for the detection of the *JAK2* mutation^9–12^. Thus, the prevalence of *JAK2* p.V617F with a ≥ 1% allele burden ranges between 0.1 and 0.5%^10–13^. However, no data is currently available on the prevalence of *JAK2* p.V617F in individuals with increased Hb or Htc, and an effective diagnostic approach has not yet been established.

To address this problem, we developed and validated a sequential two-step-algorithm screening test based on the prevalence of *JAK2* p.V617F in individuals with elevated levels of haemoglobin or haematocrit according to WHO 2016 criteria.

## Methods

### Study design and sample collection

This study was approved by the Institutional Review Board of Hospital Ramón y Cajal, Spain. Informed consent was obtained from *JAK2* p.V617F-positive patients in accordance with the Declaration of Helsinki. All *JAK2* p.V617F-positive patients were contacted. Standard diagnostic work-up was performed in accordance with relevant guidelines and regulations. The need for informed consent from *JAK2* p.V617F-negative individuals was waived by the approving ethics committee. None of the authors had access to the identity of *JAK2* p.V617F-negative individuals during data analysis.

The study was divided into two phases: a first Phase, which involved the development of an algorithm based on the prevalence of *JAK2* p.V617F in individuals with elevated Hb or Htc; and a second Phase where the algorithm was validated in an independent cohort. Blood samples were collected in EDTA tubes and analysed in a CELL-DYN Sapphire analyser (Abbott). The following parameters were measured in all samples: haemoglobin (Hb), haematocrit (Htc), leukocytes, neutrophils, platelets, mean corpuscular volume (MCV), mean corpuscular haemoglobin (MCH), and red cell distribution width (RDW).

#### Phase 1

A total of 15,366 blood samples were prospectively analysed in seven non-consecutive days. The samples that met the 2016 WHO criteria for Hb or Htc (males with Hb > 16.5 g/dL or Htc > 49%, and females with Hb > 16 g/dL or Htc > 48%) were selected for subsequent *JAK2* p.V617F mutation screening. ROC curves were used to estimate the sensitivity and specificity of leukocyte count, neutrophil count, platelet count MCV, RDW, MCH, and MCHC for detecting the *JAK2* p.V617F mutation. The variables with the best diagnostic accuracy were included in a sequential two-step-algorithm.

#### Phase 2

The two-step-algorithm was validated in blood samples from an independent cohort of 15,298 individuals. Only the samples that met the criteria of the two-step-algorithm were tested for *JAK2* p.V617F.

*JAK2* p.V617F was also tested in 300 unselected samples obtained in an outpatient setting in primary care centres to determine the prevalence of *JAK2* p.V617F clonal haematopoiesis in our environment.

### JAK2 p.V617F mutation screening

Genomic DNA was extracted from peripheral blood samples by the Chemagic MSM-I automated magnetic bead method. *JAK2* p.V617F screening was performed using a novel, allele-specific multiplex PCR assay. In this assay, we achieved the specific amplification of a 135-bp amplicon only in carriers of the c.1849G > T PV mutation by using primers 5′-GCATTTGGTTTTAAATTATGGAGTATGCT-3′ and 5′-ACACCTAGCTGTGATCCTGAAACTGA-3′. We simultaneously amplified a 291-bp genomic fragment encompassing *JAK2* exon 16. This was an internal control to rule out false negative results due to PCR failure by using primers 5′- GGCTTGAACATACTAAATGCTCCAGTA-3′ and 5′-AAGGAAAATTAACAACATGCCCTTTAC-3′. PCR was carried out using the following process: a cycle of denaturation at 94 °C for 2 min; five touchdown cycles of denaturation at 94 °C for 30 s; annealing for 30 s at 65 °C for the first cycle; a 1 °C reduction per cycle; and extension at 72 °C for 30 s; 25 cycles of denaturation at 94 °C for 30 s; annealing at 60 °C for 30 s; extension at 72 °C for 30 s; and a final extension step of 72 °C for seven minutes. The multiplex reaction with both primer pairs took place at a final concentration of 1.5 mM MgCl_2_ with Fast Start Taq DNA polymerase (Roche). PCR products were resolved in a TapeStation 2200 microfluidics electrophoresis device (Agilent).

In our assay, a threshold of mutational burden was established for the reliable detection of the p.V617F allele. To such purpose, we analysed serial dilutions of a control sample with a known p.V617F allele burden. It was confirmed that our assay consistently detected samples with mutational burdens ≥ 1% as positive for *JAK2* p.V617F.

All positive results from the multiplex assay were confirmed by quantitative real-time PCR in the Laboratory of Molecular Biology of Hospital 12 de Octubre. As previously published, samples with an allele burden > 0.71% were considered positive^14^.

### Statistical analyses

Univariate analysis was performed to assess the relationship between the variables analysed and the result of the *JAK2* test. Since all parameters, except for cell-count values (leukocytes, neutrophils and platelets), followed a normal distribution, differences were assessed using a parametric test. Student *t*-test was used for quantitative variables, whereas differences between categorical variables were assessed by the Chi-squared test. In relation to cell-count variables, a non-parametric test (Mann–Whitney U) was used. All *p* values < 0.05 were considered significant. All data were analysed using SPSS 20.0 (IBM corp.) statistical software.

## Results

### Phase 1

**Figure 1:**
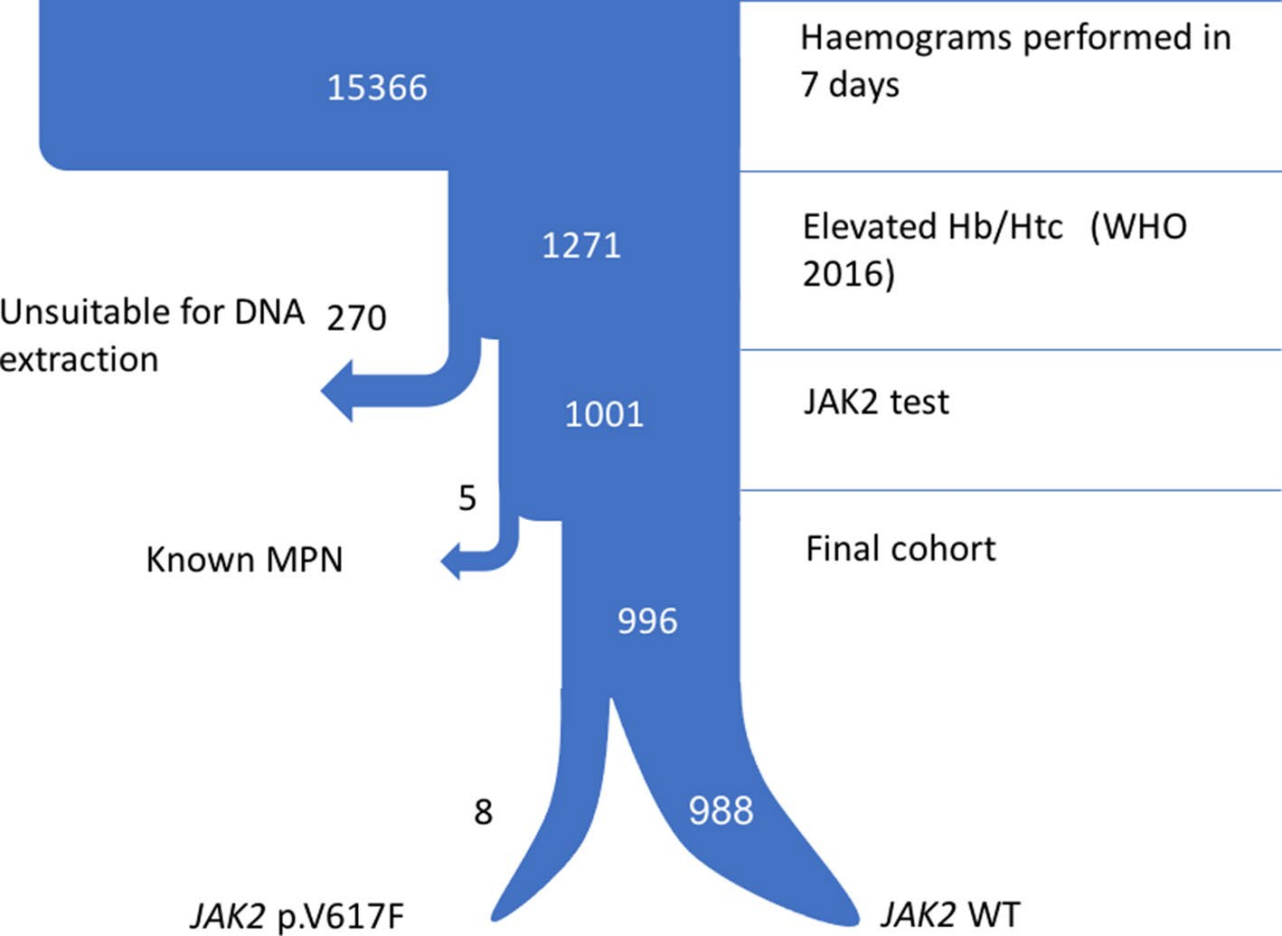
Step 1 sample flowchart. This figure contains a diagram of the sample selection process. *WHO* World Health Organization, *MPN* myeloproliferative neoplasm, *WT* wild type.

A total of 15,366 blood samples were analysed, of which 1271 (8.3%) showed elevated Hb or Htc levels according to WHO 2016 criteria, and were selected for *JAK2* testing. As many as 1001 of these samples were tested for *JAK2* p.V617F, of which 13 were positive (1.3%). The clinical records of the 13 *JAK2*-positive cases were reviewed, of which 5 were excluded because they corresponded to patients with a known myeloproliferative neoplasm. Therefore, the final prevalence of the *JAK2* p.V617F mutation in the 996 patients who met WHO Hb or Htc criteria was 0.8% (8/996). A sample flowchart is shown in Fig. 1.

The mean and median values for all the parameters studied in each group are shown in Table 1. The exclusion of 275 samples (270 were unsuitable for DNA extraction and 5 had a previous diagnosis of myeloproliferative neoplasm) had no impact on the characteristics of the groups (Table 1).

**Table 1:** Characteristics of study groups.

	All (N = 15,366)	WHO (N = 1271)	JAK2 (N = 996)
Age	60.55 (0–104)	57.83 (14–100)	58.17 (14–100)
Hb (g/dl)	13.43 (4.1–21)	16.5 (13.4–21)	16.5 (13.4–21)
Haematocrit (%)	40.76 (12.3–65)	50.63 (36.4–65)	50.52 (36.4–65)
WBC (× 10^9^/L)	7.79 (0–254)	8.36 (2.2–43.5)	8.25 (2.2–27.1)
Neutrophils (× 10^9^/L)	4.63 (0–160)	4.75 (0.9–40.9)	4.63 (0.9–22.4)
Platelets (× 10^9^/L)	233.15 (2–1096)	223.35 (37–734)	224.41 (69–734)
MCV (fL)	91.59 (52–139)	92.73 (69–119)	92.56 (69–112)
RDW	13.43 (10–37)	12.98 (11–22)	12.92 (11–22)
MCH	30.21 (15–45)	30.26 (22–47)	30.26 (22–47)
MCHC	32.98 (25–50)	32.65 (25–46)	32.70 (25–46)

*WHO* individuals meeting World Health Organization 2016 Hb or Htc criterion, *JAK2* patients in which JAK2 p.V617F was studied, *Hb* haemoglobin, *WBC* white blood cells, *MCV* mean corpuscular volume, *RDW* red cell distribution width, *MCH* mean corpuscular haemoglobin, *MCHC* mean corpuscular haemoglobin concentration.

### Algorithm development process

In order to identify the markers that could be useful to identify patients with the *JAK2* p.V617F mutation, a univariate analysis was performed according to the mutational status of *JAK2* (Table 2).

**Table 2:** Univariate analysis according to *JAK2* mutational status.

	*JAK2* p.V617F (n = 8)	JAK2 WT (n = 988)	p-value
**Sex**			
Female	1 (16.7%)	203 (20.5%)	0.816
Male	5 (83.3%)	787 (79.5%)	
Age	69.5 (18.3)	58 (9.6)	0.079
Haemoglobin	16.5 (1.2)	16.5 (0.8)	0.925
Haematocrit	51.3 (3.2)	50.5 (2.1)	0.317
WBC^b^ median (p.25;p75)	10.6 (8.2;11.6)	7.8 (6.6;9.2)	0.022
Neutrophils^b^ median (p.25;p75)	6.4 (4.7;7.8)	4.2 (3.3;5.4)	0.015
Platelets^b^ median (p.25;p75)	324 (272.5;489)	217 (183;253)	< 0.001
MCV	89.1 (4.7)	92.6 (4.6)	0.033
RDW	15.3 (3.1)	12.9 (1.2)	< 0.001
MCH	28.6 (1.7)	30.3 (1.9)	0.015
MCHC	32.1 (1.1)	32.7 (1.4)	0.247

*WBC* white blood cells, *MCV* mean corpuscular volume, *RDW* red cell distribution width, *MCH* mean corpuscular haemoglobin, *MCHC* mean corpuscular haemoglobin concentration.

^a^T-Student or Chi-square as appropriate.

^b^Non parametric U-Mann–Whitney.

We found that patients positive for the *JAK2* p.V617F mutation had statistically significant (p < 0.05) higher levels of leukocytes, neutrophils, platelets and RDW, and lower levels of MCV and MCH, as compared to *JAK2* p.V617F-negative patients (Table 2). There was a tendency in patients with *JAK2* p.V617F to be older, although differences did not reach statistical significance (p = 0.079).

For the variables with statistically significant differences, we calculated the area under the ROC curve (AUC) and the optimal cut-off points that maximize the sensitivity and specificity of the test (Youden index) (Table 3).

**Table 3:** Area under de ROC curve and optimal cut-off point*.*

	AUC	Optimal cut-off	Sensibility	Specificity
WBC	0.72	9.92	0.63	0.81
Neutrophils	0.79	5.98	0.75	0.83
Platelets	0.86	248.5	1.00	0.72
MCV	0.50	79.55	1.00	0.01
RDW	0.80	13.05	1.00	0.60
MCH	0.51	25.40	1.00	0.02

*AUC* area under the curve, *WBC* white blood cells, *MCV* mean corpuscular volume, *RDW* red cell distribution width, *MCH* mean corpuscular haemoglobin.

**Figure 2:**
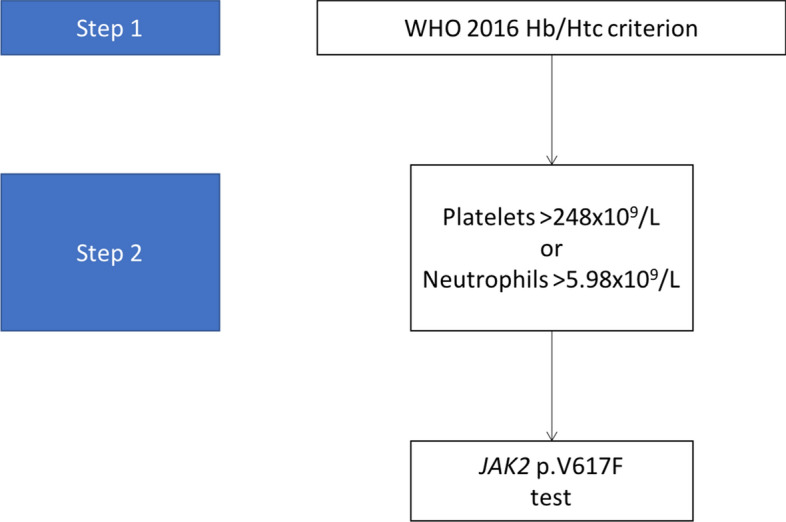
The “two-step” algorithm. This figure shows the algorithm based on data obtained in Step 1.

Of interest, neutrophils, platelets and RDW showed a high AUC (> 0.75). With a neutrophil cut-off of 5.98 × 10^9^/L, sensitivity and specificity were 75% and 83%, respectively. A platelet cut-off of 248.5 × 10^9^/L and a RDW cut-off of 13.05 showed 100% sensitivity. From this data, we developed a screening algorithm based on thresholds for platelets and neutrophils, given their statistical, clinical, and biological significance (Fig. 2).

### Phase 2

**Figure 3:**
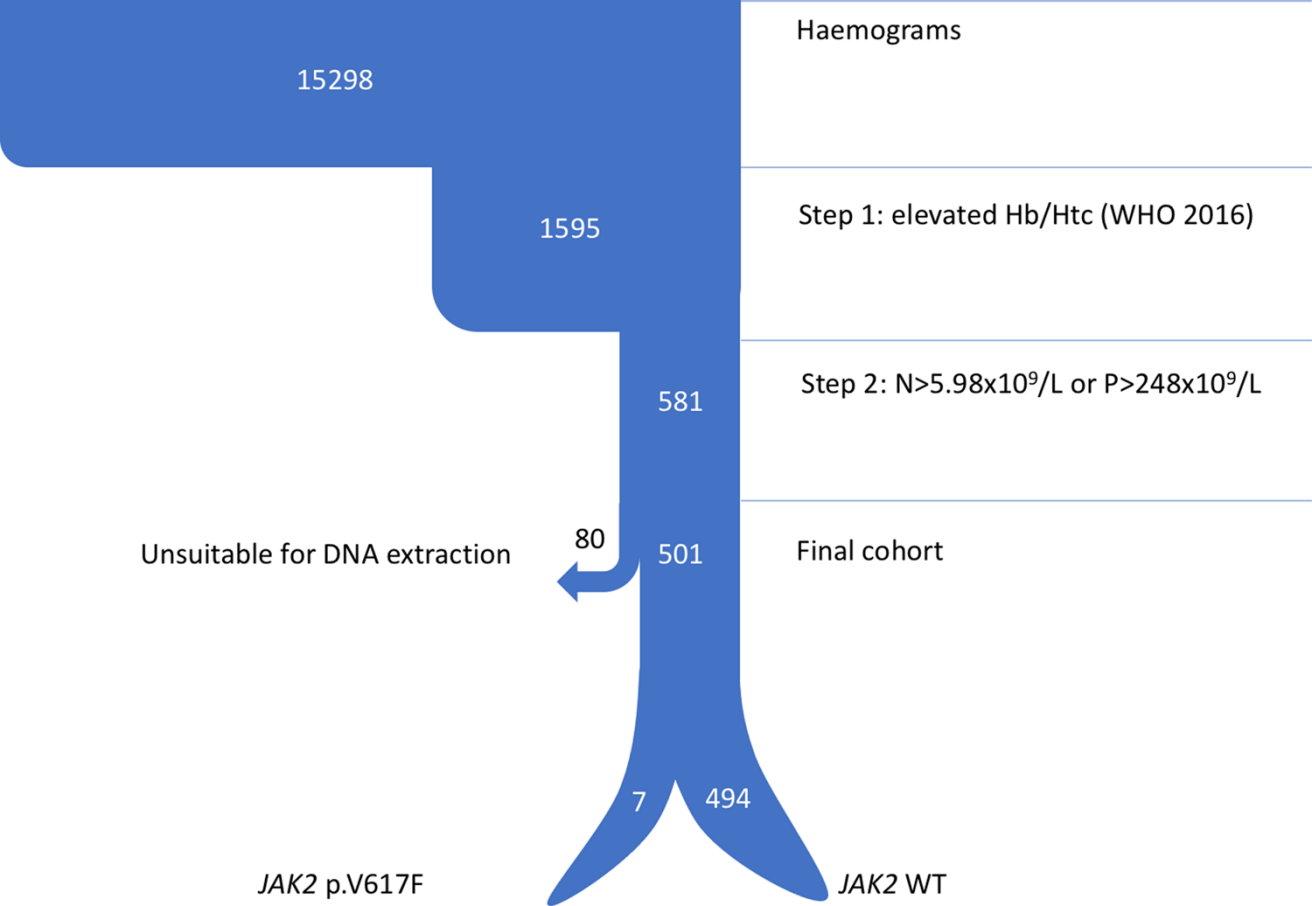
Step 2 sample flowchart. This figure contains a diagram of the samples selected in the different steps of the algorithm. *N* neutrophils, *P* platelets, *WHO* World Health Organization, *WT* wild type.

The proposed algorithm was prospectively validated in an independent cohort composed of 15,298 individuals. A total of 1595 (10.4%) patients exhibited elevated Hb or Htc levels according to the 2016 WHO criteria, of whom 581 passed to step 2 (3.8% of the total) and were selected for *JAK2* p.V617F testing. Of the latter, 80 were not suitable for DNA extraction. The *JAK2* p.V617F mutation was detected in seven of the 501 patients tested, which accounts for 0.04% of the total cohort, 0.4% of patients with elevated Hb or Htc levels, and 1.2% of the patients who fulfilled the two-step algorithm. None of these patients had a previous diagnosis of myeloproliferative neoplasm. A flowchart of samples is shown in Fig. 3.

A detailed description of the characteristics of the patients who passed Steps 1 and 2 is provided in Table S1 of online Supplementary material.

To confirm the prevalence of *JAK2* p.V617F mutation in our environment, samples from 300 unselected individuals were assayed for p.V617F, with 1 positive result that corresponded to a patient with a previous diagnosis of myeloproliferative neoplasm. Therefore, the prevalence rate was at least < 1/300.

## Discussion

The current WHO diagnostic criteria for Polycythaemia Vera include Hb and Htc values below the threshold established in the definition of erythrocytosis. This raises the question of whether all these patients should be tested for the *JAK2* p.V617F mutation. The results of this study show that an average of 1500 blood samples would be eligible every week for *JAK2* p.V617F testing, with a rate of positive results as low as 0.4%. This protocol involves an excessive workload and our results highlight that other criteria are needed for the selection of samples eligible for *JAK2* p.V617F screening.

**Figure 4:**
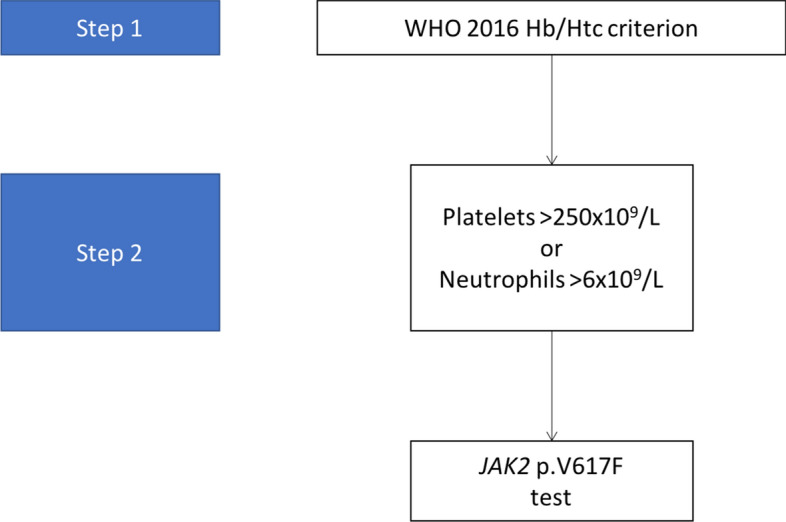
Simplified two-step algorithm. This figure shows a simplified version with rounded threshold values.

In the present work, we have designed a two-step algorithm that would reduce *JAK2* p.V617F testing to 500 samples per week. The proposed algorithm is based on blood count parameters that would increase pre-test specificity. Given that *JAK2* p.V617F-positive diseases cause myeloproliferation and PV is associated with iron deficiency secondary to erythropoiesis, we selected parameters that identified either of the two phenomena. Based on these parameters and their optimal cut-off points, we built a two-step algorithm. The first step involves the identification of males with Hb > 16.5 g/dl or htc > 49% or females with Hb > 16 g/dl or htc > 48%. The second step consists of identifying among the selected patients those who had either neutrophils > 5.98 × 10^9^/L or platelets > 248.5 × 10^9^/L. However, to facilitate the use of the algorithm, we rounded thresholds to 6 × 10^9^/L for neutrophils and 250 × 10^9^/L for platelets (Fig. 4).

Finally, the “two-step” algorithm was prospectively validated in an independent cohort of patients. However, the rate of positivity of *JAK2* p.V617F in this cohort was only 1.2%, a value too low to be used in routine practice, unless massive *JAK2* p.V617F screening platforms are developed.

Others approaches have been used in retrospective studies. Rumi et al. suggested testing for *JAK2* p.V617F in the presence of elevated Hb or Htc levels in parallel to increased neutrophils or platelets. Alternatively, if elevated Hb levels are considered alone, the threshold for screening can be increased to 17 g/dL in men^6^. However, our data showed that increasing the Hb threshold alone would not be effective, since Hb levels were below 17 g/dL in most of our *JAK2* p.V617F-positive patients (73.4%). The Canadian proposal discourages testing when other signs of myeloproliferation are present, such as platelets > 440 × 10^9^/L or neutrophils > 7 × 10^9^/L^4^. Again, if we apply that approach to our series, only 30% of our cases would meet the criteria, which indicates that the approach is suboptimal.

It is important to note that a third of patients found to be *JAK2* p.V617F-positive in the phase of prospective validation of Step 2 had suffered a cardiovascular event. Moreover, in a recent study, the presence of clonal haematopoiesis of undetermined significance in peripheral blood cells was associated with an increased risk of coronary heart disease, with the highest risk corresponding to the *JAK2* p.V617F mutation^9^. These findings support *JAK2* p.V617F screening in the general population and emphasize the need for an improved approach to the identification of potential candidates for *JAK2* p.V617F screening.

A limitation of the present study is that the prevalence of the *JAK2* p.V617F mutation was remarkably lower, as compared to previous studies. Recent data has shown that the prevalence of the *JAK2* p.V617F mutation in the general population can be as high as 3.1% when a very sensitive test is used (> 0.009%)^10^. Nevertheless, the prevalence of *JAK2* p.V617F with ≥ 1% of allele burden decreased to 0.5%, which is consistent with the results published by other groups. Our results indicate a prevalence of 0.4% of the *JAK2* V617F mutation in individuals with elevated Hb or Htc levels.

In conclusion, our data show that WHO thresholds for Hb and Htc for the diagnosis of PV should not be considered alone for *JAK2* p.V617F screening. The two-step algorithm proposed in this study improves the selection of candidates for *JAK2* p.V617F testing.

## Supplementary Information

Supplementary Information
