# Supplementary material for: Development and validation of a sequential two-step algorithm for the screening of individuals with potential polycythaemia vera
Source: Sci Rep. 2021 Jan 8;11:209. doi: 10.1038/s41598-020-80459-y (PMC7794224; doi:10.1038/s41598-020-80459-y)
Supplement: Supplementary file 1 — Supplementary Information [file 41598_2020_80459_MOESM1_ESM.pdf]

DEVELOPMENT AND VALIDATION OF A SEQUENTIAL TWO-STEP ALGORITHM FOR THE SCREENING OF INDIVIDUALS WITH POTENTIAL POLYCYTHAEMIA VERA

*Miguel Piris-Villaespesa, MD[1,2]; Alberto Álvarez-Larrán, MD, PhD[3]; Adolfo Saez-Marín, MD[1]; Claudia Nuñez-Torrón, MD[1]; Gloria Muñoz-Martin, PhD[4]; Ricardo Sánchez, MD, PhD[5]; Francisco J. del Castillo PhD[4,6,7]; Jesús Villarrubia MD, PhD[1]; Javier Lopez-Jimenez, MD, PhD[1]; Joaquin Martinez-Lopez, MD, PhD[5, 8, 9] and *Valentin Garcia-Gutierrez, MD, PhD[1,2].

[1] Haematology Department, Hospital Universitario Ramón y Cajal, Madrid, Spain.

[2] Instituto Ramón y Cajal de investigación Sanitaria (IRYCIS), Madrid.

[3] Haematology Department, Hospital Clínic, IDIBAPS, Barcelona, Spain.

[4] Translational Genomics Unit, Hospital Universitario Ramón y Cajal (IRYCIS), Madrid.

[5] Haematology Department, Hospital Universitario 12 de Octubre, Madrid.

[6] Genetics Department, Hospital Universitario Ramón y Cajal, Madrid.

[7] Centro de Investigación Biomédica en Red de Enfermedades Raras (CIBERER), Madrid.

[8] Complutense University of Madrid, Madrid

[9] Centro Nacional de Investigaciones Oncológicas, Madrid

*Corresponding author: Miguel Piris Villaespesa; mpirisv@gmail.com, +34913368368
Valentin Garcia-Gutierrez; jvalentingg@gmail.com

Abstract word count: 197

Text word count: 2596

Figures: 4

Tables: 3

Keywords: Polycythaemia Vera, *JAK2* p.V617F, algorithm, screening, myeloproliferative neoplasm

Phase	Patient	Sex	Age	Hb (g/dl)	Htcto (%)	WBC ($x10^9/L$)	Neutrophils ($x10^9/L$)	Platelets ($x10^9/L$)	MCV	RDW	MCH	MCHC	JAK2 VAF (%)	CV events
Phase 1	1	M	66	15	49,5	6,05	3,42	290	92,7	13,2	28	30,2	7,69	no
	2	M	61	16,2	50	7,83	3,42	281	95	13,1	30,8	32,5	0,92	no
	3	M	71	16,3	49,8	8,56	6,05	734	91	13,4	29,7	32,7	15,4	yes
	4	M	67	15,7	50,3	18,7	14,5	513	87,9	22,2	27,4	31,2	40,2	yes
	5	M	86	15,6	49,1	11,7	6,01	264	90,4	14,2	28,7	31,7	1,32	no
	6	F	82	17,6	54,8	9,93	7,19	465	79,6	14,4	25,5	32,1	10,8	no
	7	M	61	18,9	57,7	11,2	8,35	359	85,9	17,6	28,2	32,8	25,3	no
	8	M	62	16,6	48,9	11,5	6,65	249	90,2	14,2	30,5	33,8	14,6	yes
Phase 2	9	M	69	15,5	49,6	6,95	2,78	249	81,3	13,6	25,3	31,2	49,5	No
	10	F	62	16,2	49,6	10,1	4,96	265	95,1	11,5	31,1	32,8	100	No
	11	M	51	16,3	50,4	12,2	7,39	165	98,9	13,2	32,1	32,5	17,4	No
	12	M	69	18,1	55,3	12,2	7,17	244	88	12,8	28,8	32,8	71,1	No
	13	M	51	17,9	54,9	14,4	6,99	230	93,1	12	30,4	32,6	61,2	Yes
	14	M	77	16,3	49,4	8,63	5,72	170	95	13,3	31,3	33	9,45	Yes
	15	M	74	15,7	50,3	8,6	5,65	437	85,9	13,3	26,9	31,3	45,8	No

Supplementary Table S1: *JAK2* p.V617F patient characteristics: the results of the collected variables in the *JAK2* p.V617F patients (n=15) found among phases 1 and 2 are shown in this table. *Abbreviations: Hb: haemoglobin; WBC: white blood cells; MCV: mean corpuscular volume; RDW: red cell distribution width; MCH: mean corpuscular haemoglobin; MCHC: mean corpuscular haemoglobin concentration, VAF: variant allele fraction; CV: cardiovascular; M: male; F: female*
